# Detection of recurrent copy number alterations in the genome: taking among-subject heterogeneity seriously

**DOI:** 10.1186/1471-2105-10-308

**Published:** 2009-09-23

**Authors:** Oscar M Rueda, Ramon Diaz-Uriarte

**Affiliations:** 1 Structural and Computational Biology Programme, Spanish National Cancer Centre (CNIO), Melchor Fernández Almagro 3, 28029 Madrid, Spain; 2 Breast Cancer Functional Genomics, Cancer Research UK, Cambridge, UK

## Abstract

**Background:**

Alterations in the number of copies of genomic DNA that are common or recurrent among diseased individuals are likely to contain disease-critical genes. Unfortunately, defining common or recurrent copy number alteration (CNA) regions remains a challenge. Moreover, the heterogeneous nature of many diseases requires that we search for common or recurrent CNA regions that affect only some subsets of the samples (without knowledge of the regions and subsets affected), but this is neglected by most methods.

**Results:**

We have developed two methods to define recurrent CNA regions from aCGH data. Our methods are unique and qualitatively different from existing approaches: they detect regions over both the complete set of arrays and alterations that are common only to some subsets of the samples (i.e., alterations that might characterize previously unknown groups); they use probabilities of alteration as input and return probabilities of being a common region, thus allowing researchers to modify thresholds as needed; the two parameters of the methods have an immediate, straightforward, biological interpretation. Using data from previous studies, we show that we can detect patterns that other methods miss and that researchers can modify, as needed, thresholds of immediate interpretability and develop custom statistics to answer specific research questions.

**Conclusion:**

These methods represent a qualitative advance in the location of recurrent CNA regions, highlight the relevance of population heterogeneity for definitions of recurrence, and can facilitate the clustering of samples with respect to patterns of CNA. Ultimately, the methods developed can become important tools in the search for genomic regions harboring disease-critical genes.

## Background

Genomic DNA copy number is often variable. Some of this variability, commonly referred as copy number variations or CNVs, is naturally present in the germ line and thus heritable [1-3], whereas somatic, large-scale alterations that often characterize tumor cells are called copy number alterations or copy number aberrations (CNAs) [3-6]. These CNAs are often longer than CNVs and have been linked to other diseases in addition to cancer, such as HIV acquisition and progression, autoimmune diseases, and Alzheimer and Parkinson's disease [7-10]. The most popular current approaches for the identification of DNA copy number differences are chip- or array-based. These include SNP arrays [11-13] and array-based Comparative Genomic Hybridization (aCGH). aCGH is a broad term that encompasses oligonucleotide aCGH (Agilent, NimbleGen, and occasionally in-house oligonucleotide arrays), BAC and, less frequently nowadays, ROMA and cDNA arrays [14,15]. In addition to the array-based technologies, sequencing-based approaches [2,16-18] are also used to study CNAs. (See [3] for differences on the identification of CNVs and CNAs, and the specific challenges associated to the reliable detection of CNAs, that are due to tissue heterogeneity and contamination and uncertainty about baseline ploidy). Location of CNAs in individual samples, however, is only the initial step in the search for "interesting genes". The regions more likely to harbor disease-critical genes are those that show alterations that are recurrent among diseased individuals [15,19-21]. In this context, we can define a recurrent CNA region as a set of contiguous genes (a region) that shows a high enough probability (or evidence) of being altered (e.g., gained) in at least some samples or arrays. Unfortunately, although many methods exist for analyzing a single array (e.g., see comparisons and references in [22-25]), few papers deal with the problem of integrating several samples and finding CNA regions that are common over sets of samples. Thus, merging data from several samples to find recurrent CNA regions remains a challenge [6], both methodologically and conceptually.

Two recent reviews [4,26] highlight the main features and difficulties of existing methods. Most methods [19,20,27-30] try to find recurrent CNA regions using, as starting point, the discrete output from an aCGH segmentation algorithm in the form of the classification of every probe into gained, normal or lost. Because these methods use discretized output, they discard any available estimate of the uncertainty of these estimates; as a consequence, a gain for which there is strong evidence will have the same weight in subsequent calculations as another gain for which there is less certainty. Moreover, the majority of these methods ignore within- and among-array variability in aCGH ratios as they use a common threshold for all probes and arrays. A few other methods perform the segmentation and search for recurrent CNA regions in the same step [31-33]. The method in [33], which does not use nor returns probabilities, employs elaborate and heuristic approaches to search over possible thresholds and adjustments for multiple testing. Another two methods, [34,35], intertwine, in a complex way, biological assumptions and statistical procedures, leading to convoluted, heuristically based methods, with critical assumptions and parameters of difficult interpretation and assessment (see also [4] for a critique of the attempts to differentiate between "driver" and "passenger" mutations). In [31] copy numbers of contiguous probes as treated as independent, which is clearly biologically unrealistic. Hidden Markov Models are used by [32], but this method seems to locate recurrent probes, not recurrent regions, and the number of states is restricted to four; therefore, all the gains are grouped into a single state with a common mean, which is biologically unreasonable, and makes it impossible to differentiate between samples with moderate amplitude changes and large-amplitude changes.

In addition to the above difficulties, one the most serious problems of existing methods is the inability to find common regions over subset of samples. The majority of approaches [27,28,31,32,34-37] try to find regions that are common to all the arrays in the sample. Thus, these methods presuppose that a disease is homogeneous with respect to the pattern of CNAs. It is known, however, that for many complex diseases, such as cancer or autism [38-40], molecular subphenotypes are common. It follows that heterogeneity should be appropriately addressed [4] in studies of recurrent CNA regions. Two methods [19,33] (see also reviews in [4,26]) try to find recurrent regions defined over a subset of the samples but, in addition to not using probabilities, they depend on a resolution (or number of bins) parameter that controls the number of probes considered within region, so that, given this parameter, the method, by construction, will regard either all or none of the probes as jointly altered. But the point of searching for regions is, precisely, to identify regions for which we do not know in advance location, number of subjects, or length. Moreover, there are concerns [36] about the permutation strategy used by the above two methods to assess the statistical significance of the patterns found, as it precludes locating large aberrations. Therefore, there are currently no satisfactory approaches for addressing among-sample heterogeneity.

To further clarify and understand this problem, we can differentiate between two different scenarios. In one scenario, we consider all the samples (subjects or arrays) in the study as a homogeneous set of individuals, so we want to focus on the major, salient, patterns in the data and thus we will try to locate regions of the genome that present a constant alteration over all (or most of) the samples. This is what most existing methods for the study of recurrent CNA regions try to do. In a second scenario, we suspect that the subjects are a heterogeneous group. What we really want here is to identify clusters or subgroups of samples that share regions of the genome that present a constant alteration. In other words, we want to detect recurrent alterations in subtypes of samples when we do not know in advance which are these recurrent alterations nor the subtypes of samples. This second scenario is arguably much more common than the first one in many of the diseases where CNA studies are being conducted. In this second scenario, using an algorithm appropriate for the first scenario (one that, by construction, tries to find alterations common to most arrays) is clearly inappropriate: it does not answer the underlying biological question, risks missing relevant signals, and leads to conceptual confusion.

Existing methods, therefore, have serious limitations and it is necessary to develop new approaches that fulfill the following three major requirements. First, we want to explicitly differentiate between the two scenarios in the last paragraph. As a consequence, we want to be able to locate either regions common to most of the arrays or regions common to only a subset of the arrays. Second, we want to preserve the uncertainty in the state of a probe (probability of alteration), and we want to return probabilities, as a probability is the single most direct answer to the question "is this region altered over this set of arrays?" (a p-value does not directly answer this question, but rather provides support against a specific null hypothesis). Third, we want that the biological meaning of the regions found be immediate, which we can try to achieve by using methods that depend on few parameters of straightforward interpretation. We have developed two approaches that fulfill these criteria.

## Results

### Two different approaches for finding recurrent CNA regions

Here we provide an intuitive understanding of our two different approaches. Further details are provided below.

Our first method, **pREC-A **(probabilistic recurrent copy number regions, common threshold over all arrays), finds those regions that, over the complete set of arrays, show an average (over arrays) probability of being altered that is above a predefined threshold. When using **pREC-A **we only need to provide one threshold, *p*_*a*_, the minimal probability of alteration of a region over a set of arrays. *p*_*a *_is chosen by the researcher, but generally cannot be too stringent (e.g., will rarely be larger than 0.80) because even with a large number of arrays, only a few arrays without that alteration will prevent finding the region (as we are averaging over arrays).

Our second method, **pREC-S **(probabilistic recurrent copy number regions, subsets of arrays), identifies all common regions over subsets of arrays; alternatively, we can think of this algorithm as identifying subsets of arrays that share regions of alteration. The regions of alteration found might not be common to most arrays, but within each array in the identified subset, the regions of alteration will have a probability of being altered above a threshold (*p*_*w*_). When using **pREC-S**, therefore, the user needs to provide two thresholds, *p*_*w*_, the minimal probability of alteration of a region in every array in the selected subset, and *freq.array*, the smallest number of arrays (i.e., the smallest size of the subset of arrays) that share a common region. Here we will often use more stringent thresholds for probability (e.g., *p*_*w *_= 0.90), because those high probabilities might be attained over a highly homogeneous and small subset of arrays. We can use the output of **pREC-S **as the basis for clustering and to display patterns of groupings of arrays; an example is shown below (see "Simple numerical example: **pREC-S**").

For both methods, we will use probabilities of alteration as returned, for example, by RJaCGH [24]. RJaCGH is a Hidden Markov Model-based approach that returns probabilities of alteration of probes and segments; no hard thresholds are imposed, and thus the user decides what constituted sufficient evidence (in terms of probability of alteration) to call a probe gained (or lost). We have shown [24,25] that RJaCGH performs as well as, or better than, competing methods in terms of calling gains and losses, and the relative advantage of the method increases as the variability in distance between probes increases. It is essential to understand that the probabilities that we use are not the marginal probabilities of alteration but the joint probabilities of alteration of a region of probes (see details in "Computation of the joint probability of an arbitrary sequence of probes in an array"). Our approach incorporates both within-and among-array variability (as it is based on the hidden process of alterations and uses the probability of every probe in every array): we use the information on the certainty of each call of gain/loss (i.e., the probability) in all computations of recurrent CNA regions. Therefore, our approach is qualitatively different from using the same threshold over all probes and arrays. See further details below. Moreover, using probabilities of alteration (instead of magnitude of change), in addition to differentiating between evidence of alteration and estimated fold change, prevents inter-array differences in range of *log*_2 _ratios and tissue mixture to get confounded with evidence of alteration. Finally, note that we use at most two parameters and that their biological meaning is immediate: probability of alteration, and number of samples that share an alteration (the later only needed for **pREC-S**).

### Algorithms

Before we can develop algorithms for the two approaches, **pREC-A **and **pREC-S**, we will need to develop methodology that will allow us to: 1) compute the joint probability of alteration of an arbitrary sequence of probes; 2) combine that probability over arrays. The first two parts of this section detail this machinery before showing the details of the algorithms. For the rest of this section, please bear in mind that we are always referring to probabilities of alteration, and never to p-values. We are working on a Bayesian framework and are estimating posterior probabilities; we are not conducting hypothesis tests.

#### Computation of the joint probability of an arbitrary sequence of probes in an array

To find altered regions, that is, sets of contiguous probes, we have to compute the joint probability of alteration for a sequence of probes. In other words, we need to compute, for each array *i *= 1,... *r*, the probability that a subset of consecutive probes is, for example, gained (the problem for losses is equivalent). That is, if we denote as *S*_*i *_the state of probe *i *and with 1 the state 'gain', we are interested in *P*(*S*_*j *_= 1,..., *S*_*j*+*p *_= 1) for a subset of contiguous *p *probes. (Note that, strictly, we can find *P*(*S*_*j *_= 1,..., *S*_*j*+*p *_= 1) also for the case of non-contiguous probes, but this scenario is unlikely to be of any interest in the search for recurrent CNA regions.)

Using RJaCGH (or other methods) we can compute the probability for every probe to belong to any of the states of gain and to any of the states of loss. The problem of these probabilities is that they are marginal probabilities: they are the probability of the event of an alteration of a probe without considering the alteration of other probes, in particular of neighboring probes. But the states of the probes are not independent [24], and thus the probability of alteration of a region (within an array) can not be computed simply as the product of the probability of the individual probes.

With HMM it is customary to obtain the most likely path of hidden states using the Viterbi algorithm which returns the maximum a posteriori sequence (MAP). The Viterbi algorithm, however, does not return any distributional statements about the states of the path [41]. It is straightforward, however, to compute the marginal probabilities of the state of a probe or the joint probabilities of an arbitrary sequence of probes, because the sequence of hidden states conditioned on the parameters of the HMM is a Markov Chain [41]. For instance, we could compute the probability that the first three probes are jointly gained: *P*(*S*_1 _= 1, *S*_2 _= 1, *S*_3 _= 1) using straightforward conditional probabilities as *P*(*S*_1 _= 1)*P*(*S*_2 _= 1|*S*_1 _= 1)*P*(*S*_3 _= 1|*S*_2 _= 1), and these conditional probabilities can be computed by backward-smoothing. The problem is that the classification of probes or regions into states given by these two approaches (Viterbi and backward-smoothing) does not always coincide, leading to inconsistencies. For example, we might obtain a sequence of hidden states with maximum marginal probabilities that is not the same as we obtain with Viterbi; that sequence might even contain two consecutive altered probes that can not be jointly altered [42]. This is a common problem that can arise when using maximum likelihood approaches to HMM.

To avoid these problems, we can use, as RJaCGH does, Markov Chain Monte Carlo (MCMC) instead of Maximum Likelihood (ML). With MCMC, however, we can not average the conditional probabilities obtained through the MCMC iterations, because that would break the Markovian property [43], as we are averaging over different runs with (potentially) different values for the model parameters (as new values for the parameters are drawn at each iteration of the MCMC). For instance, suppose we want to compute the probability that the first three probes are jointly gained: *P*(*S*_1 _= 1, *S*_2 _= 1, *S*_3 _= 1). We cannot compute *P*(*S*_1 _= 1)*P*(*S*_2 _= 1|*S*_1 _= 1)*P*(*S*_3 _= 1|*S*_2 _= 1), with those conditional probabilities obtained by averaging over the multiple MCMC runs. What we can do, instead, is compute the probability of an alteration for any arbitrary sequence as the frequency of that sequence being altered in the MAPs from each of the MCMC draws. For the previous example, we would count in how many MAPs (from Viterbi) we found *S*_1 _= *S*_2 _= *S*_3 _= 1. We must note that, in this case, we are not obtaining the real distribution of the hidden states per se, but the distribution of the hidden states as members of the maximum a posteriori hidden sequence [44]. That is, we do not sample from the distribution of the hidden states, but from the distribution of the MAP. This is coherent with the classification method used with just one array, as every sequence is only accounted for if it has been part of the MAP sequence, and thus this is a stronger requirement as the regions obtained have always been part of the MAP.

Finally, the above scheme can be applied both to models that assign to hidden states probabilities of being altered of either 1 or 0, and to models that assign to hidden states probabilities of being altered between 0 and 1.

#### Combining regions over arrays

Once we have computed the probability that the above region is altered, for our first algorithm, **pREC-A**, we need to know how to average over the arrays to get a probability of alteration for that region over a set of arrays. Many HMM models (RJaCGH included) will model each array with a different HMM, to reflect the fact that they can have different characteristics, such as dispersion. Thus, for each array, we have a (potentially different) stochastic process for the log-ratios. Once the data are summarized as states (gain, loss, no-change), however, they are comparable across arrays as we are using the same approach to label probes as gained/lost/not-changed. In other words, a value of *S*_*j *_= 1 has the same meaning regardless of the array. Thus, we can average directly all the probabilities for every array (the averages might be weighted if there are differences in the reliability or the precision of different arrays). Therefore, the probability that a given region of the genome is altered over a set of arrays is computed as:

(1)

where different *P*(*array*_*j*_) allow us to use different weights for different arrays (and, of course, the *P*(*array*_*j*_) are scaled, if needed, so that ∑_*j *_*P*(*array*_*j*_) = 1).

For notational convenience, when there is only one probe, we define

(2)

#### pREC-A: Finding regions with a probability of alteration of at least *p*_*a*_

The following algorithm (Table 1) finds all the regions with an average (average over all arrays) probability of alteration of at least *p*_*a*_. This algorithm is the one that is most similar to other existing approaches in objective. Notice, however, the simplicity of our algorithm, and the straightforward interpretation of its parameters. A detailed explanation of each line of the algorithm and its logic is provided in the Additional file 1.

**Table 1:** pREC-A algorithm

**1**	*Start *← 1
**2**	**while ***Start *≤ *Total Number Of Probes ***do**
**3**	*P*1 ← *P*(*S*_*Start *_= 1);
**4**	**if ***P*1 ≥ *p*_*a *_**then**
**5**	*End *← *Start *+ 1;
**6**	**while ***End *≤ *Total Number Of Probes ***do**
**7**	*P*2 ← *P*(*S*_*Start*_,..., *S*_*End *_= 1);
**8**	**If ***P*2 <*p*_*a *_**then**
**9**	**break **out of the while loop;
**10**	**else**
**11**	*P*1 ← *P*2;
**12**	*End ← End *+ 1;
**13**	**end**
**14**	**end**
**15**	UpdateRegionA(*Start, End *- 1, *P*1);
**16**	*Start *← *End*;
**17**	**else**
**18**	*Start *← *Start *+ 1;
**19**	**end**
**20**	**end**

#### pREC-S: Finding all the regions shared by at least *freq.array *arrays where each region in each array has a probability of at least *p*_*w*_

In this algorithm (Table 2) we are imposing two thresholds: 1) *p*_*w*_, the minimum joint probability, within array, for each region; 2) *freq.arrays*, the minimum number of arrays that share the alteration. Notice that *p*_*w *_in this algorithm is different from *p*_*a *_in the previous algorithm (where averaging over arrays is used). This algorithm has no equivalent in alternative methods. A detailed explanation of each line of the algorithm and its logic is provided in the Additional file 1.

**Table 2:** pREC-S algorithm

**1**	**for ***Start *← 1 **to ***Total Number Of Probes ***do**
**2**	*SetArrays_A *← *ϕ*;
**3**	**for ***array *← 1 **to ***Total Number Of Arrays ***do**
**4**	**if ***P*(*S*_*Start *_= 1|*array*) ≥ *p*_*w *_**then**
**5**	*SetArrays_A *← *SetArrays_A *∪ *array*;
**6**	**end**
**7**	**end**
**8**	**if **|*SetArrays_A*| ≥ *freq.arrays ***then**
**9**	*End *← *Start *+ 1;
**10**	**while ***End *≤ *Total Number Of Probes ***do**
**11**	*SetArrays_B *← *ϕ*;
**12**	**foreach ***candidate array ***in ***SetArrays_A ***do**
**13**	**if ***P*(*S*_*Start*_,..., *S*_*End *_= 1|*candidate_array*) ≥ *p*_*w *_**then**
**14**	*SetArrays_B *← *SetArrays_B *∪ *candidate_array*;
**15**	**end**
**16**	**end**
**17**	**if **|*SetArrays_B*| <*freq.arrays ***then**
**18**	**break **out of the while loop
**19**	**else**
**20**	**if ***|SetArrays_B| < |SetArrays_A| ***then**
**21**	UpdateRegionS(*Start, End *- 1, *SetArrays_A*);
**22**	*SetArrays_A *← *SetArrays_B*;
**23**	**end**
**24**	*End *← *End *+ 1;
**25**	**end**
**26**	**end**
**27**	UpdateRegionS(*Start, End *- 1, *SetArrays_A*);
**28**	**end**
**29**	**end**

#### Simple numerical example: pREC-A

Suppose we have fit a model to six probes and four arrays and, after using RJaCGH's model averaging, we have obtained the marginal probabilities of gain shown in Table 3. We want to use **pREC-A **with *p*_*a *_= 0.6. First, we average the probability for probe 1 for the four arrays:

**Table 3:** Simulated data example.

	**S1**	**S2**	**S3**	**S4**	**S5**	**S6**
A1	0.17	0.17	0.97	0.97	0.97	0.17
A2	0.16	1.00	1.00	1.00	0.15	1.00
A3	0.08	0.07	0.93	0.07	0.06	0.92
A4	0.16	0.16	0.99	1.00	1.00	1.00

Marginal probabilities of being gained.



As it does not reach the threshold of 0.6, S1 can not belong to a region. We do the same for S2, obtaining 0.35. For S3 the averaged probability is 0.97, so the first region will include this probe. To see if we can extend this region to the next probe, we compute for every array the joint probability of probes 3 to 4 to be gained. This probability is not shown in the table above (which shows only marginal probabilities) but is obtained as explained above (see section "Computation of the joint probability of an arbitrary sequence of probes in an array"): the relative frequency of a sequence in the MAPs from all the MCMC samples.



As it is over the threshold, we join S4 to the region.

Now we check if S5 can be joined too. We compute the joint probability of gain for the probes 3 to 5 (again, the joint probability is computed from the relative frequency of this sequence in the MAPs from all the MCMC samples):



As it does not reach 0.6, S5 will not be part of the region, so we get:

Region 1: {(S3, S4)}.

Now we keep on searching from probe 5. S5 does not have a marginal probability higher than the threshold, so it will not form any region. But S6 will:



So it will form its own region. As there are no more probes, the regions found are {(S3, S4), (S6)}.

Boundaries of regions are forced to be common over all arrays: the algorithm finds the common regions. For instance, the left boundary of the first region of gain of sample A2 is located in probes S2, whereas the boundary for all the other three samples is located in S3. Thus, S2 is excluded from the first common region: a region that spanned {(S2, S3, S4)} would not reach, over all four arrays, the required *p*_*a *_= 0.6.

#### Simple numerical example: pREC-S

We use the same data as above. We want to find all regions where at least two arrays have a joint probability of gain of at least 0.9 (note that we raise the probability threshold because we do not ask that, on average, all arrays reach it, but at least two of them do). In other words, we are using **pREC-S **with *freq.arrays *= 2 and *p*_*w *_= 0.90. Line numbers below refer to the lines in the algorithm.

We start on S1, but there is no array that reaches the threshold of 0.9 for that probe (i.e., the condition in line 4 of Table 2 is not fulfilled for any array). We iterate (line 1 of Table 2) to the next probe, S2, but the threshold is reached only in Array 2, and we imposed that there should be at least 2 arrays. Thus, condition in line 8 is not met. We iterate to the next probe, S3. Here, when we iterate over all the arrays (line 3) we find all of the arrays reach the threshold, so in line 5 we end up with *SetArrays_A *= (*A*1, *A*2, *A*3, *A*4). As the condition in line 8 is fulfilled we try to increase the region by one probe: we set *End *to S4 (line 9) and enter the "while" loop (line 10) as we are not yet at the end of the total number of probes.

After looping over all four arrays (line 12) we find that line 13 is only fulfilled for Arrays 1, 2 and 4:



Note that the last expression is obvious since *P*(*S*4 = *Gain|A*3) = 0.07.

Therefore (from the iteration over line 14) we have *SetArrays_B *= (*A*1, *A*2, *A*4). We still fulfill the condition about *freq.arrays *in line 17, but the new set of arrays contains fewer than before (line 20) which means that in the step before a region was found. We call *UpdateRegionS *so that the region ((*S*3), (*A*1, *A*2, *A*3, *A*4)) is stored, and we set *SetArrays_A *= (*A*1, *A*2, *A*4) (line 22). We increase *End *to S5 (line 24), and consider it as the end of the new possible region. Iterating again (line 12) we find



As above, this means that in the previous step we found a region (line 20 is true). Therefore, we call *UpdateRegionS *to store the region from the previous step: ((*S*3, *S*4), (*A*1, *A*2, *A*4)). We increase *End *to S6 and find

(3)

Now, the condition in line 17 is true, because only one array satisfies being over *p*_*w*_. We break out of the while loop (line 19) and we *UpdateRegionS *in line 27, so we store the region from the previous step:((*S*3, *S*4, *S*5), (*A*1, *A*4)).

We continue iterating over *Start *(line 1), so now *Start *= *S*4. Repeating the steps above we would find a first region ((*S*4), (*A*1, *A*3, *A*4)), and a second region ((*S*4, *S*5), (*A*1, *A*4)). However, when executing *UpdateRegionS*, we would find each of these regions is a subset of a previously found region (((*S*4), (*A*1, *A*3, *A*4)) of ((*S*3, *S*4), (*A*1, *A*3, *A*4)); ((*S*4, *S*5), (*A*1, *A*4)) of ((*S*3, *S*4, *S*5), (*A*1, *A*4))).

When we iterate over *Start *to *Start *= *S*5, we find only the region ((*S*5), (*A*1, *A*4)) which is again a subset of a previously found region.

Finally, we set *Start *= *S*6. We find (lines 3 and 4) that *p*_*w *_is satisfied by arrays A2, A3, A4, so we end up with *SetArrays_A *= (*A*2, *A*3, *A*4). We fullfill the requirement about *freq.arrays*, but in line 10, however, we find we are at the end of the total number of probes, so we do not enter that loop (lines 11 to 24 are skipped). We therefore call *UpdateRegions*, and add the region ((*S*6), (*A*2, *A*3, *A*4)). (Note that the call to *UpdateRegions *in line 27 with *End *- 1 is correct, since we increased *End *one position over S6 in line 9). Therefore, we end up with the regions:

Regions = {((S3), (A1, A2, A3, A4)), ((S3, S4), (A1, A2, A4)), ((S3, S4, S5), (A1, A4)), ((S6), (A2, A3, A4))}

**Figure 1:**
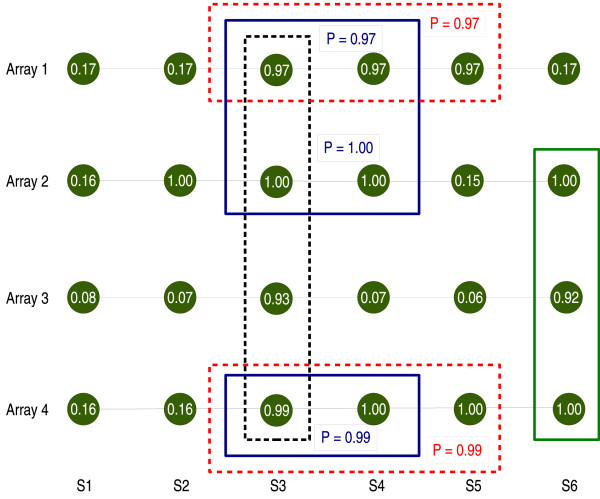
**pREC-S, simple numerical example**. Subsets of at least 2 arrays that share common regions of gain of at least 0.90 probability: *freq.arrays *= 2, *p*_*w *_= 0.90. Boxes of the same color represent the same region. In circles, the marginal probabilities of gain. In boxes, the joint probabilities.

We can see the regions obtained in Figure 1. In contrast to **pREC-A**, boundaries need not be common over arrays; with **pREC-S **differences in boundaries will lead to different subsets and different regions (for instance, that is why the common region (S3, S4) includes only samples A1, A2, A4, but not A3).

**Figure 2:**
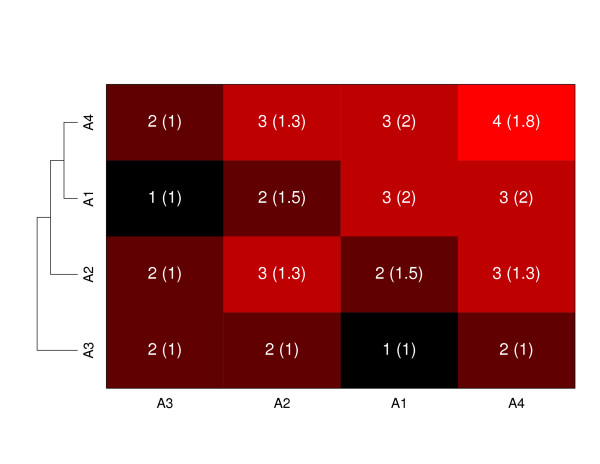
**Clustering based upon pREC-S**. Number of common regions shared by pairs of arrays. In parenthesis, the average length in probes of the regions. On the left, a dendrogram using hierarchical clustering (complete linkage) with number of common regions shared by pairs of arrays as similarity measure.

We can also use the output of this algorithm as the basis for clustering and to display patterns of groupings of arrays. We can measure similarity between two arrays as the number of common probes in recurrent CNA regions between those two arrays or, alternatively, as the number of common regions (where the same probe might belong to more than one region) between two arrays. Once similarity is measured, we can immediately apply any clustering method of our choice. An example is show in Figure 2. At this stage, clustering is mainly a device for representing patterns of similarity, since the grouping of arrays with respect to recurrent CNVs is the very output of the **pREC-S **algorithm.

### Implementation and testing

The algorithms above are part of the freely available and open-source RJaCGH R package (available from the R repositories), which uses R and C (the later, dynamically loaded from within R). For storage and efficiency reasons, we do not save directly all of the Viterbi paths (i.e., each Viterbi from each iteration of the MCMC sampler) but only the jumps in paths and the counts of different paths. This requires less storage, allows for faster access to the information and computation of the joint sequence, and of course permits reconstructing all of the sequences. The Viterbi paths are obtained as part of the regular execution of the C code for RJaCGH, saved in R as gzipped files, and read back by the C functions for **pREC-A **and **pREC-S **only once.

Execution time in all the examples of the paper is negligible: all the examples of pREC-A execute in less than 5 seconds. Execution time for pREC-S goes up to 160 seconds for the examples from [45] but less than 4 seconds for the remaining examples. (All these timings from a workstation with an AMD 280 processor running Debian GNU/Linux).

Testing was carried out by comparing the output from the algorithms with manually computed examples. Code for the examples and comparisons is included in the repository for the package .

### Examples with real data and comparison to other approaches

All the examples below were analysed with RJaCGH, which provided the probabilities of alteration. Our examples use aCGH arrays because these are three "classic" sets of data that have been analyzed before with other approaches. Our methods, however, can also be applied to other platforms, including custom and commercial oligonucleotide arrays and SNP arrays (e.g., [25]) or any other platform for which we can obtain joint probabilities of alteration. The main objective of these examples is to illustrate the range of analysis that can be performed.

#### Colorectal cancer example (Nakao et al.): direct application of pREC-A

Nakao et al. [46] analyze 125 colorectal tumors. They apply a segmentation method based on a threshold and then find common regions of alteration studying the frequency of alterations. Rouveirol et al. [28] apply both of their algorithms for minimal common regions to the same data. As shown in the Additional file 1, using **pREC-A **with a threshold of 0.35, we find basically the same regions of alteration, and most of the reported differences come from regions with a probability (or frequency, in the case of [46]) in the limit of 35%. The only remarkable case is the gain in 11q which has a much lower probability in our analysis, probably because that alteration is based on a single BAC and the segmentation analysis used in [46] is based on a threshold and therefore is more likely to be affected by outliers.

#### Colorectal cancer example (Douglas et al.): comparing probability of alterations between groups using pREC-A

**pREC-A **can also be used to compare the probability of alteration between groups of samples. Douglas et al. [47] present data from 37 primary cancers. Seven show microsatellity instability (MSI) and 30 show chromosomal instabillity (CIN). (For a definition of genetic alterations, see [48]). They call alterations using a threshold-based method and compare their frequency between the two types using a chi-square statistic. van de Wiel and van Wieringen [49] analize the same data using a dimension reduction technique (CGHRegions) after segmenting the data with DNACopy [50]. They then use a Wilcoxon test with FDR correction for the difference between the two levels.

We have used a threshold of *p*_*a *_= 0.50 to find the common regions of gain/loss and have then compared the probability of alteration in those regions for the two groups of samples. We have obtained a total of 21 regions of gain and 11 of loss, shown in Additional file 1 - Figure S2. Next, for every region found above we computed the joint probability of alteration for each of the 30 arrays of class CIN and the seven arrays of class MSI and, by region, we calculated the absolute value of the difference in mean probability between the MSI and CIN groups. To assess the significance of this statistic, we used a permutation test (randomly permuting the MSI and CIN labels and recomputing the absolute value of the difference in mean probability) to obtain a two-sided p-value. Then, we applied the FDR method [51] for multiple testing correction (to account for the multiple testing arising from comparing multiple regions). The regions found significantly different (at 0.05 level) between groups are listed in Additional file 1 - Table S3, where we also provide further details about the differences with the results in [47] and [49]. Our results are largely coincident with those in [47] and [49]. Some regions mentioned in [47] (a two-clone region in chromosome 8, a 29-clone region in chromosome 18, and the p arm of chromosome 20) are not detected by us as these are regions with probability of alteration just below 0.50. There are differences with the method of [49], CGHregions, in the location of the breakpoints: CGHregions is a dimension reduction method that simplifies the complexity of the sample profiles, which probably leads to a larger imprecision in the location of region boundaries.

#### Breast cancer example (Pollack et al.): pREC-S and homogeneity index

Pollack et al. [45] analyze data from 44 breast tumors and 10 cancer cell lines. They search for common regions of alteration and then compare the frequency of aberrations in each arm of every chromosome as a function of other variables such as tumor grade, estrogen receptor (ER) and TP53 mutations. Rouveirol et al. [28] also analyze these data. We have applied our second method, pREC-S, to the 44 tumors to examine if there is any similarity in the alterations shared by the groups of arrays defined by those variables. We have computed common regions of at least 0.50 probability of alteration (Gains or Losses) shared by at least two arrays (i.e., *freq.array *= 2, *p*_*w *_= 0.50).

To compare our approach with the results of [45], and to gain more insight on the patterns of recurrent CNA regions and their relationship to the other three variables (tumor grade, ER, TP53), we have defined a simple statistic to measure within-group homogeneity of recurrent CNA regions. Let *Y*_*ij *_be the number of probes that array *i *and array *j *have altered in common, *k *a group of arrays (typically, with some common characteristic), *n*_*k *_the number of different pairs of arrays in a given group *k *and *n_k *the number of different pairs formed by arrays in group *k *and arrays in a different group. Let us define



**Figure 3:**
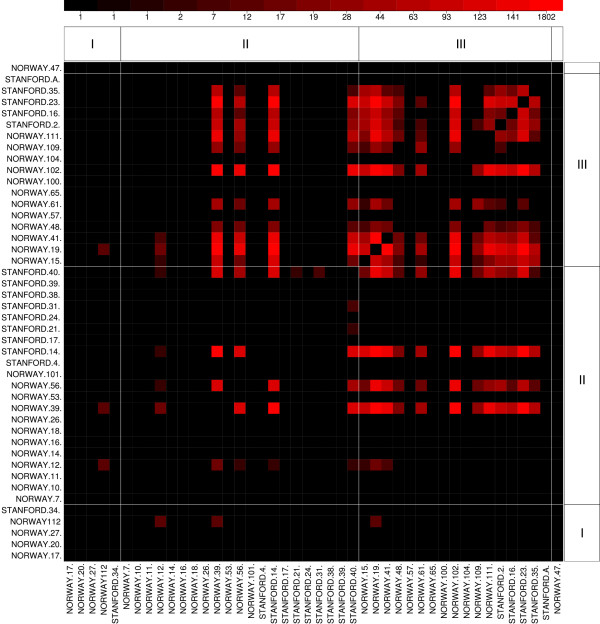
**Chromosome 8 from the Pollack et al. example**. Number of regions of gain with at least 0.50 probability shared by at least two arrays (i.e., pREC-S, *freq.arrays *= 2, *p*_*w *_= 0.50). The arrays are ordered according to tumor grade. Arrays with grade III share many more alterations between them than the other arrays. Four arrays with grade II share the same gains in copy number with tumors of higher grade, so they are probably related. There is one array unidentified.

That is,  is the average number of common altered probes between two arrays of group *k*, and  is the average number of common altered probes between one array of group *k *and other in a different group. We define the proportion of common alterations shared by the group *k *as /. This index measures the homogeneity of the genomic alterations within a subset of arrays compared to the alterations shared with arrays of other group. If this index is greater than 1, the arrays of this group share more alterations between themselves than arrays of different groups do. If this index is 0, no alterations are shared between any two arrays in the group. A value of *8 *means that no alteration is shared between arrays of this group and others. We can compute this index for the groups defined by the three variables tumor grade, ER, and TP53 mutations; this is shown in the Additional file 1 - Tables S4 to S6). Those tables allow us to easily discern chromosomes that are very homogeneous with respect to shared alterations; for instance, gains in chromosomes 4 and 5 and losses in chromosome 8 are very homogeneous in the estrogen receptor negative samples (Additional file 1 - Table S4). We can display the patterns of similarity graphically, as is done in Figure 3, where we have ordered the arrays by tumor grade and show the number of common alterations for chromosome 8. Our results are not easy to compare with [45], because they define the regions and compare subgroups at chromosome arm resolution, while our method works at probe resolution.

Furthermore [45] consider every chromosome arm as altered or not without taking into account the number of altered probes in it.

To further understand the pattern of similarities, instead of comparing subgroups according to the number of alterations, we can analyze how homogeneous each group is over the whole genome (not chromosome by chromosome, as in previous tables). This is shown in Table 4. When we divide arrays according to tumor grade, Grade I and Grade III show high homogeneity within groups, meaning that the alterations are consistent in arrays within those grades. Arrays of grade II, however, show much more heterogeneity, sharing many aberrations with arrays of Grade I and/or Grade III. This is an indication that arrays of Grade II can be classified in one of the other two groups according to the pattern of alterations. Figure 2 provides a graphical illustration: four arrays of Grade II are very similar to the arrays of Grade III.

**Table 4:** Alterations in Pollack et al. [45], genomewide.

		**pREC-S (Homogeneity index)**
ER	Positive	0.75
	Negative	1.12

p53	Wild Type	0.67
	Mutant	1.23

Grade	I	1.21
	II	0.56
	III	1.40

The homogeneity index, , is computed over the whole genome, not chromosome by chromosome, as done in previous tables.

## Discussion

We have developed two very different approaches for finding regions of recurrent, or common, copy number alteration. The lack of gold standards and the current non-existence of an unambiguous definition of what a region of recurrent CNA is [6], as well as the unique and qualitatively different nature of our methods from previous ones, make it difficult to compare performance, but at the same time highlight the relevance of our methods for current and future studies of CNA, their relation to phenotypic variation, and their usage for subject clustering.

The two methods we have developed share that they use as input probabilities of alteration and return probabilities. Regardless of whether the input probabilities are obtained from our RJaCGH method [24] or some other approach, it can be argued that probabilities are much better suited to the task at hand than p-values or discrete classifications into "gained", "lost", "not changed". By using probabilities as input, we incorporate uncertainty in the estimates of copy number states. By returning probabilites and using probabilities throughout all the analysis, the user can decide the appropriate thresholds (or, even, modify them depending on context) and define distances between arrays that incorporate the strength of evidence in favor of alteration. Precisely because of the conceptual simplicity of using probabilities, we can approach within a unified framework both questions related to "unsupervised problems" (e.g., identify subsets of regions that are common to subsets of arrays) and to "supervised problems" (e.g., measure how different two groups of arrays are with respect to recurrent regions of alteration). This unified approach is unique to our methods, and not shared by any others.

Our first method, **pREC-A**, searches for general, broad patterns of common gains (or losses) over all the samples in the study. This is the approach which is most similar to previous ones. This method is well suited to comparing pre-defined groups of samples. By its very nature (e.g., that an overall pattern is identified by a mean probability larger than a threshold) this method can only detect regions for which there is at least moderate evidence (medium probability) of alteration over almost all samples, or very strong evidence (high probability) of alteration over an important fraction of the samples. Thus, it is easy to miss regions that are present with very high probability in a small subset of the samples. As well, mixing in the same sample very heterogenous groups will tend to smooth out the evidence of alteration, so that few common regions will be found. Alternatively, if there are very different sample sizes (different number of arrays) in the different heterogenous groups, the detected common regions will often be a subset of the common regions among the most abundant group. These features can be controlled to answer the specific study questions. First, as equation 1 shows, it is easy to weight different arrays differently, so as to increase the influence of some arrays in the final analysis. Moreover, if we know in advance that there are different subgroups of samples, we can use **pREC-A **independently in the different subgroups; for instance, when we have already subdivided the subjects in the study into homogeneous groups with respect to disease (e.g., [52]), and want to locate recurrent CNA regions common to most samples within a subgroup and possibly different from other subgroups. Finally, as our last example with the data of [47] shows, a user that understands these features of **pREC-A **can employ this algorithm to highlight the differences between subgroups and how these change as we modify the minimum required threshold for the probability of alteration. In particular, note the easy formulation of a permutation-based test for identifying the differences in the probabilities of alteration of regions between subgroups. This type of approach might be even more useful when two or more suspected subgroups are compared against a larger, reference group. The main advantages of this algorithm are that it is most similar to previous approaches, has a simple interpretation in terms of global patterns across most of the samples, and requires the specification of only one parameter. Thus, **pREC-A **will often be the method of choice if we are trying to relate major, global, recurrent patterns of CNA to variations in phenotype or to differentiate between subroups of samples. In contrast to **pREC-A**, the second method, **pREC-S**, can detect small subgroups of samples with respect to common alterations, without being adversely affected by averages over arrays or differences in number of samples in different subgroups. Moreover, different subgroups can be detected with respect to different alterations. **pREC-S**, therefore, addresses a common and distinct need that arises in any study of CNA with heterogeneous samples.

As seen in the results, this second algorithm allows us to elegantly approach some of the questions in the second example (breast cancer example, [45]). First, the derivation of a specially tailored statistic, , to answer the relevant questions in this study is straightforward. More importantly, the second algorithm finds homogeneous subgroups, with respect to alterations, and these differences are associated with differences in three other markers (estrogen receptor status, TP53 mutation, tumor grade; see Additional file 1). In other words, **pREC-S **finds CNV that differentiate between groups. It must be emphasized that **pREC-S **has been applied to the complete set of data after specifying that the within-array probability of alteration be larger than 0.5 (i.e., *p*_*w *_= 0.50) and that these regions be shared among, at least, two arrays (i.e., *freq.array *= 2), but the algorithm is blind to the "labels" of the arrays regarding the other markers (estrogen receptor, TP53, grade). Therefore, **pREC-S **allows us to find CNVs that differentiate between known groups (as in this case), but its systematic usage also opens the door to finding patterns of CNA that might differentiate between previously unknown groups. Moreover, there is no need for the association recurrent CNA regions-marker to be similar among different markers, specially since, as explained above, different subgroups of arrays can be detected with respect to different CNA recurrence patterns. These are features unique and characteristic of **pREC-S**, compared to all the alternative available methods.

We suggest that **pREC-S **is the method of choice when there is unknown heterogeneity among arrays in CNAs, and when we want to relate possibly non-identical subsets of samples, defined in terms of recurrent patterns of CNA, to phenotypic variation. Moreover, routine use of **pREC-S **even with apparently homogeneous groups of samples might help discover possible subtypes of diseases that might generate novel hypothesis or uncover previously unknown heterogeneities.

**pREC-S **is also a key method for clustering. Integrative studies that combine CNV data with other data (e.g., mRNA, SNP) often use clustering of subjects based upon the CNA data (e.g., [53,54]). The problem of most of these approaches is that, when clustering based upon the CNA data (either the gain/loss calls or the smoothed data), the measure of distance or similarity used ignores that some of the data show strong serial dependence (probes next to each other) whereas some of the data (e.g., probes in different chromosomes) are independent. Thus, in most cases the distance computed is likely to introduce serious distortions in the true distances among subjects (see also [29,55,56]). This problem is in addition to the aforementioned issues of not integrating variability and uncertainty in the gain/loss calls or smoothed means. In contrast, by using a biologically motivated and probabilistically based approach to CNA common regions, such as **pREC-S**, it will be possible to construct distance metrics and, therefore, clustering approaches, that make full usage of CNA data when searching for groups of subjects. Fully developing a method for clustering based upon CNA data is outside the scope of this paper, but we have presented a simple example to motivate further work.

Moreover, an additional distinct feature of our methods is that both **pREC-S **and **pREC-A **have at most two parameters of straightforward biological interpretation (probability of alteration, number of samples that share the alteration). An added advantage of the type of input and output used by our methods is that probabilities allow researchers to modify thresholds as needed, and to easily (and intelligibly) examine the sensitivity of results to changes in thresholds.

Furthermore, as both methods are based on a Hidden Markov Model (HMM) with no restrictions on the number of states [24], we can use models involving an arbitrary number of states of gain and loss. The HMM (probabilistically) assigns probes to hidden states, but it is up to subsequent analysis to assign those states to specific or interesting "copy number states". This allows us to keep the two different concepts of "amplitude (or magnitude) of change" and "evidence of alteration" separate. Moreover, it is also immediate to restrict finding common regions to alterations above a certain threshold of amplitude or that belong only to a subset of states so that we can focus only on alterations of a certain type (e.g., only the largest hidden states of gain in a model with three hidden states for gain).

Finally, note that the problem we have been addressing is the location, *de novo*, of recurrent CNA regions. A different set of problems is using pre-existing information about regions that show copy number polymorphism to inform the search for rare copy number variants [57,58]. Likewise, another very different set of problems is the usage of previously identified variable regions in tests of association between copy number variation and disease [59-61]. These are, however, sufficiently related objectives, and methodological and conceptual advances in any one set of approaches could be highly beneficial for the other two sets of problems.

## Conclusion

We have developed methods for finding regions of copy number alteration (CNA) common or recurrent over several arrays. Our methods have an immediate and intuitive biological interpretation, and incorporate both within- and among-array variability. Reanalysis of several data sets in the literature show that our methods can indeed recover patterns previously found but can also uncover additional patterns. Moreover, probabilities allow researchers to modify thresholds as needed, and to easily examine the sensitivity of results to changes in thresholds. In addition, the examples show how it is straightforward to derive tailored statistics and summary measures to answer specific research questions. The development of these two distinct algorithms highlights a key idea that has often been neglected: recurrent or common CNAs can refer to very distinct patterns in a group of samples, specially concerning heterogeneity among arrays and probability of alteration. We expect that these two algorithms will help advance efforts to standardize definitions of recurrent or common CNA regions, and ultimately the search for genomic regions harboring disease-critical genes.

## Authors' contributions

OMR developed the statistical model, participated in the programming and conducted all of the analysis. RD-U conceived the original HMM model and participated in model development and programming. Both authors wrote, read, and approved the final manuscript.

## Supplementary Material

Additional file 1 Supplementary Material for "Detection of recurrent copy number alterations in the genome: taking among-subject heterogeneity seriously". A PDF file with further details on the algorithms and tables and comments on the Examples. Click here for file
