# Supplementary material for: Detection of recurrent copy number alterations in the genome: taking among-subject heterogeneity seriously
Source: BMC Bioinformatics. 2009 Sep 23;10:308. doi: 10.1186/1471-2105-10-308 (PMC2760535; doi:10.1186/1471-2105-10-308)
Supplement: Additional file 1 — Supplementary Material for "Detection of recurrent copy number alterations in the genome: taking among-subject heterogeneity seriously". A PDF file with further details on the algorithms and tables and comments on the Examples. [file 1471-2105-10-308-S1.PDF]

Supplementary Material for "Detection of Recurrent Copy Number Alterations in the Genome: taking among-subject heterogeneity seriously"

Oscar M. Rueda, Ramon Diaz-Uriarte
Structural and Computational Biology Programme
Spanish National Cancer Centre (CNIO)
Melchor Fernández Almagro 3, 28029 Madrid, Spain

1 Detailed explanation of the two algorithms

1.1 pREC-A: Finding regions with a probability of alteration of at least p_a

The following algorithm finds all the regions with an average (average over all arrays) probability of alteration of at least p_a. After the algorithm we provide a detailed explanation.

```
1  Start ← 1
2  while Start ≤ TotalNumberOfProbes do
3      P1 ← P(S_Start = 1);
4      if P1 ≥ p_a then
5          End ← Start + 1;
6          while End ≤ TotalNumberOfProbes do
7              P2 ← P(S_Start, ..., S_End = 1);
8              if P2 < p_a then
9                  break out of the while loop;
10             else
11                 P1 ← P2;
12                 End ← End + 1;
13         UpdateRegionA(Start, End − 1, P1);
14         Start ← End;
15     else
16         Start ← Start + 1;
```

Algorithm 1: pREC-A algorithm

The search for common regions starts on the first probe of every chromosome. If the average probability of alteration over arrays fulfills the p_a criterion (line 4) we examine if we can add probes to this region, until no further probes can be added to the region, which is equivalent to $P2$ falling below p_a (line 8). If the probe we considered as *Start* does not fullfill p_a, the next probe is considered as starting probe (line 16).

The function `UpdateRegionA` (called in line 13) adds a region to the set of regions already stored. `UpdateRegionA` records the first and last probes of the region (*Start* and $End - 1$) and the average probability of the region ($P1$, as computed in line 3 or $P2$ as computed in line 7). This function can only be called if at least the probe *Start* fulfills the p_a criterion (as the call is inside the "If" condition in line 4). We can call `UpdateRegionA` either if we are at the end of a chromosome (so there are no further probes to consider for extending a region: line 6 is not satisfied) or if the probe we just considered for addition to the region results in the average probability of the region ($P2$) to drop below p_a (line 8). The rest of the algorithm is mostly in charge of appropriately updating *Start*, *End*, $P1$, and $P2$, so that we can directly call `UpdateRegionA` (line 13) with the same arguments and without further conditional checks. Note that calling `UpdateRegionA` with $End - 1$ (and not *End*) is what we want to do to ensure that the correct last probe of a region is recorded, regardless of whether we reach line 13 from line 8 or from exiting the while loop (line 6).

Computationally, when finding $P2$ (line 7), and for a given *Start*, we do not need to repeatedly compute $P2$ over all probes of a region: it is much faster to simply update the $P2$ probability as we add one probe at a time at the end of the region (i.e., as we increase *End*).

Line 14 ensures that, when we cannot add any probes to a region (because the probability falls below p_a), the probe that will be considered as *Start* candidate for the next region is the one immediately following the *End* of the last accepted common region. As a consequence, this algorithm ensures that a probe that has a marginal probability higher that the threshold will always be part of a region (at least it will be a region itself), but does not uniquely define the regions (uniqueness is guaranteed for probes). For example, suppose we are interested in finding regions of gain of at least 0.90 probability. We can have the following situation with three probes:

$$
\begin{aligned}
P(S_1 = 1) &= 0.95 \\
P(S_1 = 1, S_2 = 1) &= 0.90 \\
P(S_1 = 1, S_2 = 1, S_3 = 1) &= 0.89 \\
P(S_3 = 1) &= 0.95 \\
P(S_2 = 1, S_3 = 1) &= 0.90
\end{aligned}
$$

Our algorithm would return two regions, $\{S_1, S_2\}$ and $\{S_3\}$. But the regions $\{S_1\}$ and $\{S_2, S_3\}$ are also valid. Accounting for these effects computationally will slow down the algorithm and, biologically, it is of no relevance because all three probes are always included in the set of regions.

Of course, the joint probability of all regions returned by this algorithm is not necessarily larger than the threshold p_a: each region has a probability of at least p_a, but this does not guarantee that, jointly, all regions have a probability of at least p_a.

This algorithm is the one that is most similar to other existing approaches in objective. Notice, however, the simplicity of our algorithm, and the straightforward interpretation of its parameters.

1.2 pREC-S: Finding all the regions shared by at least $freq.array$ arrays where each region in each array has a probability of at least p_w

We are imposing two thresholds: 1) p_w, the minimum joint probability, within array, for each region; 2) $freq.arrays$, the minimum number of arrays that share the alteration. Notice that p_w in this algorithm is different from p_a in the previous algorithm (where averaging over arrays is used).

```
1  for Start ← 1 to TotalNumberOfProbes do
2      SetArrays_A ← φ ;
3      for array ← 1 to TotalNumberOfArrays do
4          if P(S_Start = 1|array) ≥ p_w then
5              SetArrays_A ← SetArrays_A ∪ array;
6      if |SetArrays_A| ≥ freq.arrays then
7          End ← Start + 1;
8          while End ≤ TotalNumberOfProbes do
9              SetArrays_B ← φ;
10             foreach candidate_array in SetArrays_A do
11                 if P(S_Start, ..., S_End = 1|candidate_array) ≥ p_w then
12                     SetArrays_B ← SetArrays_B ∪ candidate_array;
13             if |SetArrays_B| < freq.arrays then
14                 break out of the while loop
15             else
16                 if |SetArrays_B| < |SetArrays_A| then
17                     UpdateRegionS(Start, End − 1, SetArrays_A);
18                     SetArrays_A ← SetArrays_B;
19                 End ← End + 1;
20         UpdateRegionS(Start, End − 1, SetArrays_A);
```

Algorithm 2: pREC-S algorithm

The logic of this algorithm is very similar to that of pREC-A, above. The function `UpdateRegionS` (called in lines 17 and 20) adds a region to the set of regions already stored. Adding a region means storing the first probe of the region ($Start$), the last probe of the region ($End - 1$), and the arrays that compose the region (those in $SetArrays\_A$). (Because of the way that End and $SetArrays\_A$ are updated, End and $SetArrays\_A$ are always the correct arguments to this function). The function `UpdateRegionS`, however, must check that the region to be added is not a subset of some previously added region. Suppose in the run that started with probe $S2$ we found the region $((S2, S3, S4), (A1, A2))$. Now, in the run that starts with probe $S3$ we find the region $((S3, S4), (A1, A2))$; obviously, the newly found region is simply a completely contained subset of the previously found region, and we should not add this

newly found region as a new region.

The conditions in lines 4 and 11 refer to one of the conditions of the algorithm: an array can only be considered part of a common region if the probability of the given sequence of probes (starting at *Start* and ending at *End* or, in the one-probe case, starting and ending at *Start*) is larger than p_w. Likewise, the conditions in lines 6 and 13 refer to the second condition: at least $freq.arrays$ arrays must fulfill that the sequence has a probability larger than p_w.

Line 16 represents the condition where the number of arrays that fulfill the condition when we add a probe decreases. In other words, at step t, with $End = Start + t$, we had a set of arrays that fulfilled p_w. As soon as we add a new probe (i.e., "stretch" the region by one probe, so we are at step $t + 1$ with $End = Start + t + 1$), at least one array no longer satisfies p_w. This means that at step t we had one common region over a set of arrays to which we cannot add another probe. Therefore, as soon as the number of arrays in *SetArrays_B* becomes smaller than *SetArrays_A*, we know we found a common region in the previous step, and we have to update the set of regions.

Line 18 is needed to allow capturing subsequent decreases (if there were any) in the number of arrays that meet the condition as we keep enlarging the region by adding probes.

We only reach line 20 if we exit the while loop (line 8). This can happen in two ways: either because we no longer fulfill $freq.arrays$ (line 15) or if there are no further probes to consider because we are at the end of the chromosome. In the first case, we know we have to add the sequence in the previous iteration (so the argument $End - 1$ is correct, as it was *End* which lead to failing the condition in line 13). In the second case, we have to add the sequence up to the last probe (and again $End - 1$ is the correct argument as we increased *End* in line 19).

Analogous to what happened in **pREC-A**, computing $P(S_{Start}, \ldots, S_{End} = 1|candidate\_array)$ (line 11) requires only an update, not computing the probability of the complete set of probes each time.

In any specific implementation, it is not necessary to explicitly do assignments as in lines 2 and 9. In our current C implementation, we use two additional variables (one for the vector that represents *SetArrays_A* and one for the vector that represents *SetArrays_B*) that tell us how many valid elements there are in each set, and we only access and use up to those valid elements. Likewise, the set union operation as in lines 5 and 12 can instead be implemented as an assignment to a specific position of a vector. Similar comments apply to line 18. For instance, we could rewritte lines 4 and 5 as:

$valid\_elements \leftarrow 0$;
if $P(S_{Start} = 1|array) \geq p_w$ **then**
    $valid\_elements \leftarrow valid\_elements + 1$;
    $SetArrays\_A[valid\_elements] = array$;

valid_elements is also the cardinality of the set. (Note that in C and other languages that index arrays starting at 0 we would increase *valid_elements* after the assignment to *SetArrays*).

This algorithm has no equivalent in alternative methods.

2 Examples

2.1 Colorectal cancer example (Nakao et al.): direct application of pREC-A

Table 1 and the frequency plot of alterations (Figure 1) show the results using pREC with a threshold of 0.35. Most of the differences between our results and those of Nakao *et al.* (2004) and Rouveirol *et al.* (2006) come from regions with a probability (or frequency, in the case of Nakao *et al.* (2004)) in the limit of 35%. The only remarkable case is the gain in 11q which has a much lower probability in our analysis, probably because that alteration is based on a single BAC and the segmentation analysis used in Nakao *et al.* (2004) is based on a threshold and therefore is more likely to be affected by outliers. In Table 2 we show the results with a threshold of 0.5 at BAC resolution. The results are also similar to Rouveirol *et al.* (2006), but they only provide a small excerpt in their paper, so direct comparisons are difficult to make.

2.2 Colorectal cancer example (Douglas et al.): comparing probability of alterations between groups using pREC-A

In Figure 2 we show the common regions with at least 0.50 probability of alteration and the joint probability for those regions for the two groups.

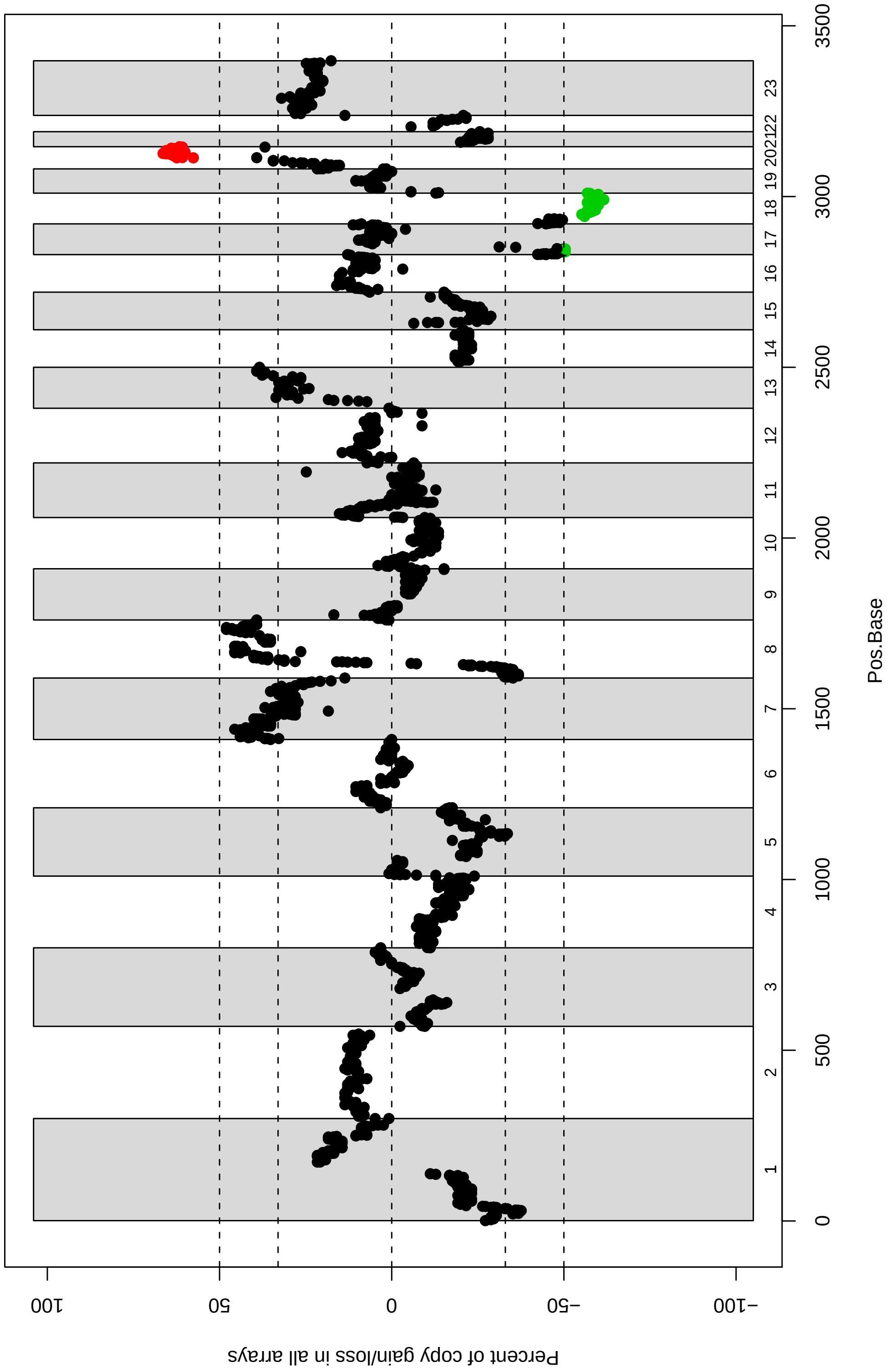

Figure 1: Frequency plot of the alterations in 125 colorectal tumor samples in Nakao et al. The red dots show gains found in more than 50% of the samples, and the green dots losses in more than 50%. The dotted lines show the 33% and the 50% frequency.

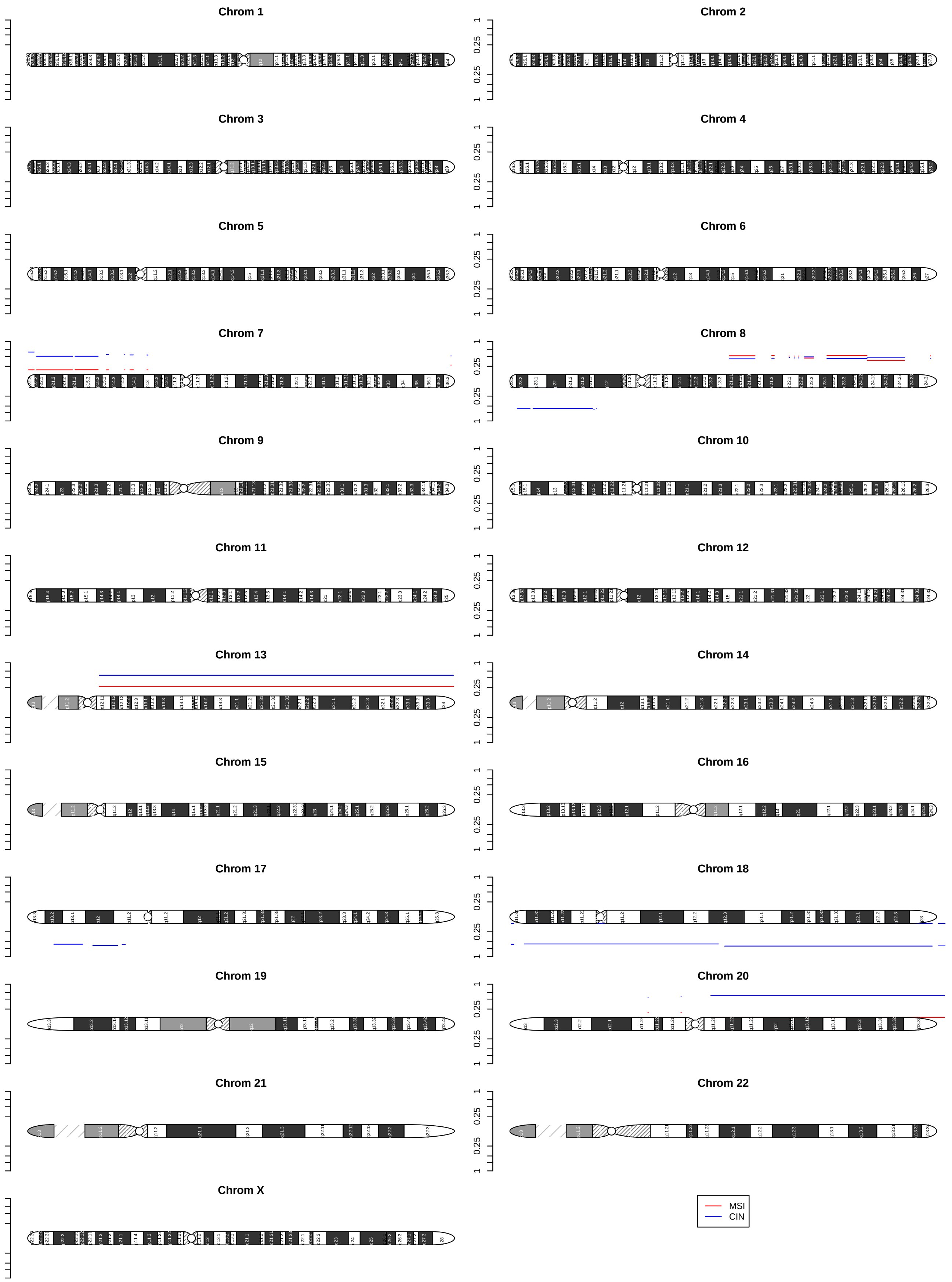

Figure 2: Joint probability in MSI and CIN alterations for the Common Regions of at least 0.5 probability (i.e., pREC-A, $p_a = 0.5$). Along the abscissa, for each chromosome, the position; the coordinate indicates the probability, with values below 0 indicating loss, and above 0 gain.

Gain		Loss	
Nakao et al.	pREC-A	Nakao et al.	pREC-A
7p	7p	-	1p
7q	7q	5q	5q
8q	8q	8p	8p
11q	-	17p	17p
-	13q	18	18
20q	20q	21q	-

Table 1: Common regions in Nakao et al. Nakao *et al.* (2004) Results using pREC-A, $p_a = 0.35$. This analysis shows results at chromosomal arm resolution.

Chrom.	Start (Mb)	End (Mb)	#Probes	Prob.	Alteration
20	32.33	32.33	2	0.5976	Gain
20	32.718	46.643	19	0.5003	Gain
20	47.321	60.461	29	0.5948	Gain
20	63.878	63.878	1	0.5026	Gain
20	64.021	65	2	0.6219	Gain
17	7.518	7.518	1	0.5002	Loss
17	8.17	8.17	1	0.5054	Loss
17	9.118	9.118	1	0.5038	Loss
17	10.004	16.581	5	0.5002	Loss
18	3.731	5.014	3	0.5019	Loss
18	6.251	6.251	1	0.5026	Loss
18	9.188	10.75	2	0.5038	Loss
18	10.862	11.925	3	0.5119	Loss
18	13.559	13.559	1	0.5087	Loss
18	14.77	58.594	15	0.5009	Loss
18	62.332	90	18	0.5438	Loss

Table 2: Common regions in Nakao et al. Nakao *et al.* (2004) pREC-A, $p_a = 0.50$. Analysis at BAC resolution.

As explained in the paper, for every region found above we computed the joint probability of alteration for each of the 30 arrays of class CIN and the seven arrays of class MSI and, by region, we calculated the absolute value of the difference in mean probability between the MSI and CIN groups. To assess the significance of this statistic, we used a permutation test (randomly permuting the MSI and CIN labels and recomputing the absolute value of the difference in mean probability) to obtain a two-sided p-value. Then, we applied the FDR method Benjamini & Hochberg (1995) for multiple testing correction (to account for the multiple testing arising from comparing multiple regions). The regions found significantly different (at 0.05 level) between groups are listed in Table 3: Douglas *et al.* (2004) report differences between both groups in gain of chromosome 20, loss of 18q and the short arm of chromosome 17 and loss of 8p. Our regions do not include the complete chromosome 20 because the p arm is gained with probability less than 0.5. We found also a difference in all of chromosome 18, but Douglas *et al.* (2004) report some losses in certain clones in the MSI group that we have not found. The rest of the regions we found are reported in Douglas *et al.* (2004) as common regions of alteration but with no difference. The later could be related to the higher precision that our method gives, but Douglas *et al.* (2004) do not provide details about frequency of those regions.

Comparing these results to van de Wiel & van Wieringen (2007), we first find that almost all of the regions of van de Wiel & van Wieringen (2007) are discovered with our method, but the length or the location of the breakpoints sometimes differ, as explained in the main text. Second, two regions in their paper, a small loss region of only two clones in the 8th chromosome and a big region of 29 clones in chromosome 18th, are not detected by our method because the probability of loss of those regions is just below 0.50 of probability. Of course, our method allows to adjust the threshold at whichever value is considered reasonable, and to check how conclusions change with changes in the threshold. Finally, there are other regions detected by our method that show significant differences between the two groups and are not reported in van de Wiel & van Wieringen (2007), such as losses in chromosome 17 (detected in Douglas *et al.* (2004)) and gains in chromosome 7.

Chrom.	Start	End	#Probes	Prob. alteration	Alteration	p-value	FDR-adjusted p-value
7	254610	2436414	5	0.5493	Gain	0.0001	0.0002
7	3293630	16702590	19	0.5001	Gain	0.0104	0.0166
7	17587872	26210052	9	0.5020	Gain	0.0111	0.0169
7	29279112	30070392	2	0.5195	Gain	0.0042	0.0090
7	35993377	35993377	1	0.5130	Gain	0.0048	0.0090
7	38011390	39281976	2	0.5065	Gain	0.0061	0.0103
7	44307040	44727994	2	0.5045	Gain	0.0053	0.0094
13	19104448	113866204	103	0.5327	Gain	0.0278	0.0404
20	20191940	20191940	1	0.5055	Gain	0.0047	0.0090
20	25023262	25023262	1	0.5441	Gain	0.0006	0.0014
20	29402772	63589868	51	0.5535	Gain	< 0.0001	< 0.0001
8	2520596	6933218	10	0.5023	Loss	< 0.0001	< 0.0001
8	7938098	28300098	25	0.5040	Loss	< 0.0001	< 0.0001
8	28775788	28775788	1	0.5252	Loss	< 0.0001	< 0.0001
8	29649361	29649361	1	0.5135	Loss	< 0.0001	< 0.0001
17	4824380	10156678	12	0.5072	Loss	< 0.0001	< 0.0001
17	12025982	16624989	6	0.5371	Loss	< 0.0001	< 0.0001
17	17432136	18029867	2	0.5170	Loss	< 0.0001	< 0.0001
18	225168	707954	3	0.5091	Loss	< 0.0001	< 0.0001
18	2572772	37207434	41	0.5011	Loss	< 0.0001	< 0.0001
18	38298595	75324734	47	0.5531	Loss	< 0.0001	< 0.0001
18	76423282	77615559	6	0.5297	Loss	< 0.0001	< 0.0001

Table 3: Region differences in Douglas et al.Douglas *et al.* (2004). Regions that differ with respect to copy number alterations between CIN and MSI groups in the data set from Douglas *et al.* (2004). See text for details of test.

2.3 Breast cancer example (Pollack et al.): pREC-S and homogeneity index

We use here pREC-S with $freq.array = 2$, $p_w = 0.50$. As explained in the text, we have defined a simple statistic to measure within-group CNVCR homogeneity. This index measures the homogeneity of the genomic alterations within a subset of arrays compared to the alterations shared with arrays of other group. If this index is greater than 1, the arrays of this group share more alterations between themselves than arrays of different groups do. If this index is 0, no alterations are shared between any two arrays in the group. A value of ∞ means that no alteration is shared between arrays of this group and others. We can compute this index for the groups defined by the three variables tumor grade, ER, and TP53 mutations.

In Table 4 we see that the gains in chromosomes 4 and 5 and the losses in chromosome 8 are very homogeneous in the estrogen receptor negative samples. Table 5 shows that the gains in chromosomes 2 and 10 and the losses in chromosomes 17 and 21 are more homogeneous in tumors harboring TP53 mutations. Finally, Table 6 also shows differences in the pattern of homogeneity of alterations with respect to the grade of the tumor.

	Gain		Loss	
Chrom.	ER='+'	ER='-'	ER='+'	ER='-'
1	0.61	1.16	1.54	0
2	0.12	0	1.93	0.17
3	46.67	0	2.77	0.51
4	0	3.43	0.66	0.65
5	0.21	2.72	0.46	1.37
6	0.12	2.1	0.39	1.4
7	2.27	0.09	0.25	0.85
8	0.5	1.54	0.22	1.52
9	0.55	1.7	1.68	0.11
10	3.62	0	2.06	0
11	2.82	0.19	0.26	0.07
12	11.03	0	0.83	0
13	0.94	0.21	0.38	1.26
14	0.99	0	0.85	0.59
15	0.71	0.3	∞	0
16	2.18	0.34	0.72	1.25
17	0.55	1.55	2.14	0.9
18	0.27	1.73	2.61	0
19	1.04	0	1.22	0
20	0.8	0.94	0.58	1.49
21	0.66	0.68	0.92	0.56
22	0.48	0.67	2.09	0
X	1.56	0	0.53	0.38

Table 4: Alterations in Pollack et al. Pollack *et al.* (2002) by Estrogen Receptor Values shown: $\bar{Y}_k/\bar{Y}_{-k}$

	Gain		Loss	
Chrom.	p53='Wt'	p53='Mutant'	p53='Wt'	p53='Mutant'
1	0.83	0.86	3.96	0.5
2	0	36.71	0.82	0.66
3	0.45	0.79	0.2	1.51
4	0	2.82	0.73	0.73
5	0.17	1.44	1.73	0.51
6	0.12	1.29	0.88	0.74
7	0.55	1.24	1.17	0.5
8	0.3	2.31	0.35	0.96
9	1.46	0.79	0.67	1.13
10	0.2	33.18	1.65	0.25
11	0.63	0.84	0.78	0.14
12	1.51	0	0.14	0.51
13	5.23	0.48	0.63	0.88
14	0.89	0.6	0.04	3.76
15	0.06	1.01	0.26	0.24
16	0.52	1.51	2.32	0.34
17	0.43	1.72	0.63	8.23
18	0.19	2.21	2.24	0.28
19	0.58	0.49	1.14	0.26
20	0.64	1.24	0.52	1.51
21	0.66	0.91	0.35	1.9
22	0.25	1.78	1.6	0.07
X	0	0.71	0.37	1.12

Table 5: Alterations in Pollack et al. Pollack *et al.* (2002) by TP53 mutations. Values shown: $\bar{Y}_k/\bar{Y}_{-k}$

Chrom.	Gain<br>Grade I	Grade II	Grade III	Loss<br>Grade I	Grade II	Grade III
1	1	0.39	1.04	0	1.07	0.28
2	0	0.03	0	4	0.2	0.53
3	0	2.14	0.02	0.86	0.6	2
4	0	0	0	0	0.65	1.12
5	0	0.01	3.45	0.89	0.57	0.75
6	0	1.59	0.42	1.33	1.06	0.72
7	2.58	0.22	0.75	0	1.52	0.41
8	0	0.47	2.47	0	0.74	0.64
9	5.36	0.32	0.49	0.81	1.06	0.81
10	0	0	1.67	1.87	1.08	0.05
11	0.16	0.47	1.48	0	0.86	0.12
12	0	0.83	0.09	6.33	0.44	0.81
13	1.91	0.14	0.82	0.15	0.34	1.85
14	0	0.6	1.54	0.79	0.25	1.38
15	0	1.05	0.5	0	0.55	0
16	0	0.77	1.32	0	1.4	1.15
17	0	0.29	3.56	0.64	0.11	1.89
18	1.12	0.22	1.47	0.57	1.1	0.47
19	0	1.02	0.36	2.71	2.86	0
20	0	0.39	2.49	0	0.55	1.11
21	1.14	0.68	0.66	1.57	0.71	0.86
22	0	0.39	0.7	0	1.1	0.18
23	0	0	∞	1.83	1.57	0.1

Table 6: Alterations in Pollack et al. Pollack *et al.* (2002) by tumor grade. Values shown: $\bar{Y}_k/\bar{Y}_{-k}$

References

Benjamini, Y. & Hochberg, Y. (1995) Controlling the false discovery rate: a practical and powerful approach to multiple testing. *J. Roy. Statist. Soc. Ser. B,* **57**, 289–300.

Douglas, E., Fiegler, H., Rowan, A., Halford, S., Bicknell, D., Bodmer, W., Tomlinson, I. & Carter, N. (2004) Array comparative genomic hybridization analysis of colorectal cancer cell lines and primary carcinomas. *Cancer Res.,* **64** (14), 4817–4825.

Nakao, K., Mehta, K., Fridlyand, J., Moore, D., Jain, A., Lafuente, A., Wiencke, J., Terdiman, J. & Waldman, F. (2004) High-resolution analysis of dna copy number alterations in colorectal cancer by array-based comparative genomic hybridization. *Carcinogenesis,* **25** (8), 1345–1357.

Pollack, J., Sorlie, T., Perou, C., Rees, C., Jeffrey, S., Lonning, P., Tibshirani, R., Botstein, D., Borresen-Dale, A. & Brown, P. (2002) Microarray analysis reveals a major direct role of dna copy number alteration in the transcriptional program of human breast tumors. *Proc Natl Acad Sci U S A.,* **99** (20), 12963, 12968.

Rouveirol, C., Stransky, N., Hupé, P., La Rosa, P., Viara, E., Barillot, E. & Radvanyi, F. (2006) Computation of recurrent minimal genomic alterations from array-cgh data. *Bioinformatics,* **22**, 2066–2073.

van de Wiel, M. A. & van Wieringen, W. (2007) Cghregions: dimension reduction for array cgh data with minimal information loss. *Cancer Informatics,* **2**, 55–63.
